# *Trichoplusia ni* Transcriptomic Responses to the Phytosaponin Aglycone Hederagenin: Sex-Related Differences

**DOI:** 10.1007/s10886-024-01482-1

**Published:** 2024-03-05

**Authors:** Yinting Chen, Christine Lafleur, Ryan J. Smith, Diljot Kaur, Brian T. Driscoll, Jacqueline C. Bede

**Affiliations:** 1 Department of Plant Science, McGill University, 21,111 Lakeshore, Ste-Anne-de-Bellevue, QC H9X 3V9 Canada; 2 Department of Animal Science, McGill University, 21,111 Lakeshore, Ste-Anne-de-Bellevue, QC H9X 3V9 Canada; 3 Natural Resource Sciences, McGill University, 21,111 Lakeshore, Ste-Anne-de-Bellevue, QC H9X 3V9 Canada

**Keywords:** Antifeedant, Cytochrome P_450_, Hederagenin, Saponin, Sex-specific, Transcriptomics, Toxicity

## Abstract

Many plant species, particularly legumes, protect themselves with saponins. Previously, a correlation was observed between levels of oleanolic acid-derived saponins, such as hederagenin-derived compounds, in the legume *Medicago truncatula* and caterpillar deterrence. Using concentrations that reflect the foliar levels of hederagenin-type saponins, the sapogenin hederagenin was not toxic to 4th instar caterpillars of the cabbage looper *Trichoplusia ni* nor did it act as a feeding deterrent. Female caterpillars consumed more diet than males, presumably to obtain the additional nutrients required for oogenesis, and are, thus, exposed to higher hederagenin levels. When fed the hederagenin diet, male caterpillars expressed genes encoding trypsin-like proteins (LOC113500509, LOC113501951, LOC113501953, LOC113501966, LOC113501965, LOC113499659, LOC113501950, LOC113501948, LOC113501957, LOC113501962, LOC113497819, LOC113501946, LOC113503910) as well as stress-responsive (LOC113503484, LOC113505107) proteins and cytochrome P_450_ 6B2-like (LOC113493761) at higher levels than females. In comparison, female caterpillars expressed higher levels of *cytochrome P*_*450*_* 6B7-like* (*LOC113492289*). Bioinformatic tools predict that cytochrome P_450_s could catalyze the oxygenation of hederagenin which would increase the hydrophilicity of the compound. Expression of a *Major Facilitator Subfamily (MFS) transporter* (*LOC113492899*) showed a hederagenin dose-dependent increase in gene expression suggesting that this transporter may be involved in sapogenin efflux. These sex-related differences in feeding and detoxification should be taken into consideration in insecticide evaluations to minimize pesticide resistance.

**Supplementary Information:**

The online version contains supplementary material available at 10.1007/s10886-024-01482-1.

## Introduction

Saponins are plant specialized metabolites important for defense against pathogens and insect herbivores, particularly in legume crops (Hussain et al. 2019; Roopashree and Naik 2019; Zaynab et al. 2021). Saponin levels, specifically oleanolic acid-derived saponins, have been correlated to resistance of the barrel medic, *Medicago truncatula*, and related plant species to caterpillar herbivory (Adel et al. 2000; Agrell et al. 2003; Cai et al. 2017). This research was performed to determine if the oleanolic acid-derived sapogenin hederagenin acts as a toxin or antifeedant, contributing to this resistance. In addition, caterpillar countermeasures to detoxify hederagenin were investigated through a transcriptomic study with special attention paid to sex-related differences in the expression of genes encoding detoxification enzymes.

The barrel medic *Medicago truncatula*, a forage crop in Australia and Mediterranean countries (Crawford et al. 1989; Howieson et al. 2000), has morphological and chemical defenses to protect itself against insect herbivores of which triterpenoid saponins are key defensive plant specialized metabolites (Bede 2020). Over 85 triterpenoid saponins are found in *M. truncatula* roots and above-ground tissues (Lei et al. 2019). From the precursor β-amyrin, triterpenoid saponins can be classified into groups depending on the aglycone: soyasaponin B and E, hederagenin, bayogenin and medicagenic acid, of which the last three are derived from oleanolic acid (Vincken et al. 2007; Moses et al. 2014). Cai et al. (2017) compared different *M. truncatula* ecotypes for resistance against herbivory by caterpillars of the beet armyworm, *Spodoptera exigua* (Hübner). Ecotypes with higher oleanolic acid-derived saponins were more resistant to caterpillar herbivory which appear to reflect antifeedant activity. In particular, foliar levels of medicagenic-, zanhic acid- and hederagenin-type saponins, are more than twice as high in the more caterpillar-resistant HM006 (F83005.5) compared to the caterpillar-susceptible HM020 (TN1.11) ecotype (Cai et al. 2017; Lei et al. 2019). Foliar tissues of the more caterpillar-resistant HM006 had a higher number of unique (hexose-hexose-hexose-hederagenin, hexose-hexenuronic acid-hederagenin) or twofold higher levels (hexose-rhamnose-hexose-hexose-hederagenin, hexose-hexose-hexenuronic acid-hederagenin, hexose-deoxyhexose-hexose-hexenuronic acid-hederagenin) of hederagenin-type saponins than the more susceptible HM020 (Cai et al. 2017; Lei et al. 2019).

Saponins can negatively affect insect herbivores in different ways (De Geyter et al. 2007). Haemolytic-type saponins, for example oleanolic acid-derivatives, can form pores in the lining of the insect gut, leading to fluid loss and eventually death (Voutquenne et al. 2002; Gauthier et al. 2009; De Geyter et al. 2012). Less haemolytic saponins, for example medicagenic acid-derivatives, form micelles that can prevent the absorption of hydrophobic nutrients, such as sterols. Since insects cannot synthesize steroidal precursors and rely on obtaining them from their diet (Behmer and Nes 2003; Lavrynenko et al. 2015), reduction of dietary lipids could impact levels of sterol-derived ecdysteroid hormones that are crucial for insect development. Saponins may also non-specifically inhibit insect gut-associated digestive enzymes through protein–saponin interactions (Ishaaya 1986).

Saponins may also act as antifeedants (Jain and Tripathi 1991; Koul 2008; Zalucki and Furlong 2017). When insects detect these compounds, they may or will not feed on the plant. The triterpenoid saponin, 3-O-[O-β-D-glucopyranosyl-(1 → 4)-β-D-glucopyranosyl]-hederagenin, found in bittercress *Barbarea vulgaris*, is a strong feeding deterrent to caterpillars of the diamondback moth, *Plutella xylostella* (Shinoda et al. 2002). Wound-induced saponins, particularly soyasaponin- and medicagenin-type saponins, are proposed to act as antifeedants to Egyptian cotton leafworm, *Spodoptera littoralis*, caterpillars (Agrell et al. 2003).

Upon ingestion, plant- and caterpillar midgut-associated glycosidases may hydrolyze the ester linkage between the sugar group(s) and the sapogenin aglycone (Liu et al. 2017; Terra et al. 2019; Lacchini et al. 2023). In *M. truncatula* roots, upon tissue damage, β-glucosidase G1 hydrolyzes the sugar from the C28 position of 3-Glc-28-Glc-medicagenic acid (Lacchini et al. 2023). Likely similar β-glycosidases that hydrolyze glycosyl ester bonds may be found in foliar tissue. As well, caterpillars secrete α-glucosidases and β-glycosidases into their midguts (Liu et al. 2018; Terra et al. 2019; Lacchini et al. 2023); presently, little is known if these enzymes are able hydrolyze the saponin ester bond linking the sugar to the aglycone. Saponins are also labile in alkali solutions releasing the sapogenin. Thus, the alkaline caterpillar gut (~ pH 9), the hydrolysis of the saponin to the aglycone may occur spontaneously (Dow, 1992).

Though some biological activity is linked to the saponin, in particular, the enhanced water solubility of the saponin is important for biological function, the sapogenin aglycone may also have biological activity. For some haemolytic saponins, after interaction with the red blood cell, hydrolysis of the glycosidic ester bonds precedes haemolysis (Segal et al. 1974; Segal and Milo-Goldzweig 1974; Voutquenne et al. 2002). Similarly, the aglycone is responsible for the antifungal cell lytic activity of some saponins (digitonin, tomatine, α-hederin) against *Rhizoctonia solani* and *Botrytis cinerea* mycelia (Segal and Schlösser 1975). The sapogenin hederagenin showed antibacterial activity against the gram positive *Bacillus subtilis* (Avato et al. 2006). The insecticidal effects of hederagenin on caterpillars of the Egyptian cotton leafworm, *Spodoptera littoralis*, was more potent than the saponin 28-*O*-β-glucopyranosyl-3-*O*-[α-L-arabinopyranosyl (1 → 2)-β-D-glucopyranosyl-(1 → 2)-α-L-arabinopyranosyl]-hederagenin (3-Ara-Glc-Ara, 28-Glc H) (Adel et al. 2000). Feeding caterpillars hederagenin or the saponin increased days to pupation and resulted in decreased pupal weight. However, in addition a decrease in fecundity (eggs laid/female) was also noted for caterpillars fed hederagenin.

How oleanolic-derived saponins protect *M. truncatula*, as toxins or antifeedants, is unclear. To understand this, we used caterpillars of the cabbage looper, *Trichoplusia ni*. These insects are adaptive specialists; they can feed on a broad range of plant hosts but prefer Brassicaceous plants, making them aggressive pests of cruciferous crops, particularly in greenhouses (Capinera 2003; Cervantes et al 2011). Brassicaceous plants, such as those in the *Barbarea* genus, protect themselves with saponins, particularly hederagenin-derived compounds (Shinoda et al. 2002). As explained above, these compounds can act as antifeedants and modify caterpillar feeding behaviour or may have direct noxious effects on the insect. However, insects have multiple strategies to reduce the impact of toxins, such as reducing penetration, target site modification and detoxification enzymes (Siddiqui et al. 2023). In particular, caterpillars often upregulate expression of gut- and Malpighian tubule-associated detoxification genes when exposed to xenobiotics (Amezian et al. 2021).

After uptake, xenobiotic detoxifications involve three phases: functionalization, conjugation and excretion (Amezian et al. 2021; Esteves et al. 2021). In phase 1, enzymes, such as esterases or cytochrome P_450_s, modify the xenobiotic either catabolizing it or making it more hydrophilic for excretion. In phase 2, molecules, such as glutathione or glycosides, can be added either directly to the xenobiotic or to the modified xenobiotic, by enzymes such as glutathione *S*-transferases (GSTs) or UDP-glycosyl transferases (UGTs), respectively, again, often to enhance excretion. In the *T. ni* genome, 199 putative detoxification genes have been identified: 108 cytochrome P_450_s, 34 GSTs and 57 α-carboxylesterases (Fu et al. 2018). Our understanding of which of these enzymes are involved in the detoxification of plant specialized metabolites and insecticides as well as the regulation of their expression is still relatively preliminary (Amezian et al. 2021).

Male and female insects may have different abilities to detoxify xenobiotics. Female caterpillars need to acquire sufficient protein at the larval stage for ovarian development and yolk production (Wheeler 1996; Wheeler et al. 2000). This is reflected in their feeding and nutrient utilization. Female *Spodoptera litura* caterpillars select a more protein-rich diet than males and utilize these proteins for growth more efficiently (Lee 2010). Considering this difference in nutrient requirements, females may invest less in detoxification enzymes prioritizing egg production over detoxification or may invest more in detoxification to ensure overall fitness. As well, more detoxification genes are associated with female sex-linked genes than males as evidenced by the higher levels of *CYP* and *GST* genes on the W chromosome of the diamondback moth, *Plutella xylostella* (You et al. 2013).

Saponins are involved in protection of many plant species against pathogens and insect herbivores (Hussain et al. 2019; Roopashree and Naik 2019; Bede 2020; Zaynab et al. 2021). Foliar levels of oleanoic acid-derived saponins, such as hederagenin-derivatives, are correlated with caterpillar deterrence (Cai et al. 2017). In vitro toxicity and antifeedant assays were performed with caterpillars of the cabbage looper, *T. ni*. Given that plant β-glycosidases may be needed to convert the saponin to the aglycone, we investigated the direct effect of the sapogenin hederagenin on the insect. As well, transcriptomic experiments were conducted to understand possible mechanisms of detoxification and if there is a sex-specific difference in detoxification-related gene expression.

## Methods and Materials

### Chemicals

Hederagenin was purchased from Cayman Chemical Company. All other chemicals, including ethanol, RNAlater™ Solution etc., were purchased from ThermoFisher Scientific.

### Insect Colony

From *Trichoplusia ni* eggs initially obtained from the Canadian Forest Service – Great Lakes Forestry Service (CFS-GLFS), caterpillars were reared on a modified McMorran-Grisdale diet and kept at 28.5 °C, 28–40% humidity, 16:8 light to dark cycle (Cai et al. 2017). Pupae were moved to an aquarium to allow adult moths to emerge and mate. Eggs were collected from a sterile mesh hung from the roof of the aquarium and used to maintain the colony. Adults were provided with a 10% honey solution (Ebling and Dedes 2015). For the experiments, when insects were third instars, males and females were separated based on the presence of yellow gonads in the males. Fourth instar caterpillars were used in the experiments.

### Determination of the Hederagenin Concentration to use in Bioassays

Hederagenin-type saponin levels range from 0.018 to 0.2 mg/g DW in *M. truncatula* (Huhman et al. 2005; Tava and Pecetti 2012; D'Addabbo et al. 2020). Based on the assumptions that *M. truncatula* leaves are approximately 80% water and caterpillars (*Spodoptera exigua*) normally eat ~ 25 mg DW of leaf material in a day (Cai et al. 2017), a caterpillar should be exposed to 25 × the measured foliar saponin (DW) levels. Therefore, 8 μg and 80 μg hederagenin added to the diet approximate the constitutive and induced levels that a caterpillar would be exposed to while feeding on *M. truncatula* plants.

### Experiment 1. Hederagenin Toxicity

Hederagenin (concentration range: 2 to 80 μg in ethanol) or ethanol (4 μL) was added to a small piece of artificial diet (~ 0.1 g) to ensure that it would be consumed in 24 h. The concentration range of hederagenin tested was reflective of levels in *M. truncatula* leaves and was limited by the solubility of the compound in ethanol (D'Addabbo et al. 2020; Tava and Pecetti 2012; Huhman et al. 2005). The diet was placed in the centre of an unsealed Petri dish and allowed to sit for 2 h to ensure that the ethanol evaporated. A male or female *T. ni* caterpillar was placed in the dish and allowed to feed. If the caterpillar consumed all the diet, artificial diet alone was added to ensure that the mortality was due to hederagenin and not lack of food. Mortality was recorded at 24 and 48 h. Mortality was determined by lack of movement when the insect was prodded with forceps. For each sex and concentration, 10 caterpillars were tested. The experiment was repeated with no toxicity observed over the concentration range tested.

### Experiment 2. Hederagenin Antifeedant Activity

In no-choice studies, 4^th^ instar *T. ni* caterpillars were given artificial diet spiked with either ethanol or hederagenin and the amount of diet eaten in 18 h determined. In a 60 mm Petri dish, a large piece of pre-weighed artificial diet (0.5 ~ 0.6 g) was placed in the middle to which ethanol or 8 μg or 80 μg hederagenin dissolved in ethanol (4μL) added. After the ethanol evaporated (2 h), a male or female caterpillar was added to the Petri dish at 16:00 and allowed to feed for 18 h in the growth chamber with settings: 28.5 °C, 28–40% humidity, 16 h light and 8 h dark cycle. At that time, any diet left in the Petri dish was collected into labeled envelopes, followed by drying in the oven at 50 °C for 3 days and then weighed in preweighed envelopes. In addition, a control without caterpillars was performed to determine the fresh weight (FW) to dry weight (DW) conversion ratio of the diet: R_(FW/DW)_ = FW/DW. In each replicate, ten caterpillars were tested per treatment (ethanol control or two hederagenin concentrations, male or female = 60 insects). Four temporal replicates were performed.

The amount of diet consumed was analyzed by two-factor analysis of variance (2-way ANOVA) (Factors: sex, concentration) (SPSS statistics ver. 28) followed by a Tukey HSD *post-hoc* test.

### Experiment 3. Effect of Hederagenin on *Trichoplusia ni* Detoxification Gene Expression.

The experimental set-up was as described in Experiment 2, except that the caterpillar was allowed to feed for 24 h. The whole gut with attached Malpighian tubules was dissected, rinsed in sterile lepidopteran saline (0.25 g NaCl, 0.768 g KCl, 1.7 g CaCl_2_•2H_2_O, 1.63 g MgCl_2_•6H_2_O, 0.476 g HEPES, 6.48 g glucose dissolved in 400 mL distilled water, pH 7.2 (Christensen et al. 1991)), placed in RNA later and stored at -20 °C until RNA extraction. A food bolus in the gut was not observed for any samples. One caterpillar was dissected per treatment (ethanol control or two hederagenin concentrations, male or female). The experiment was temporally repeated three times.

### RNA Extraction and Next-Generation Sequencing (RNA-Seq)

Total RNA was extracted from *T. ni* samples using Qiagen RNeasy Mini Kit following the manufacturer’s protocol. The gut and attached Malpighian tubules were removed from the RNA later and placed in a 1.5 mL Eppendorf tube containing lysis buffer (600 μL of 1:100 μL β-mercaptoethanol:Buffer RLT) and kept at room temperature for 3 min. Tissues were disrupted by homogenization (ProCulture™ Cordless Homogenizer Unit) using a sterile grinder that was changed between samples. Homogenized samples were centrifuged at 16,000 × *g* for 3 min to separate phases. The supernatant containing the total RNA was carefully moved into another Eppendorf tube to which ethanol (70%, 600 μL) was added followed by mixing the lysate by pipetting. The sample (600 μL) was transferred onto a RNeasy Mini spin column in a collection tube (2 mL) and centrifuged at 16,000 × *g* for 15 s. RW1 buffer (350 μL) was added to each column and centrifuged again for 15 s at the same speed and the flow-through discarded. On column DNase digestion was conducted to avoid potential DNA contamination in the sample. Samples were incubated at room temperature for 15 min. After adding RW1 buffer (350 μL) to the column, samples were centrifuged at 16,000 × *g* for 15 s. Similarly, RPE Buffer (500 μL) was added to each column and samples were centrifuged at 16,000 × *g* for 15 s. After discarding the flow-through, Buffer RPE (500 μL) was added again and samples were centrifuged at 16,000 × *g* for 2 min. Columns were placed in new collection tube and total RNA was eluted by adding RNase-free water (50 μL) followed by centrifugation at 16,000 × *g* for 1 min. To ensure a high RNA yield, the collection step was repeated twice.

Total RNA samples were submitted to Genome Québec (Montreal, Canada) who prepared libraries (NEB) followed by sequencing (Illumina NovaSeq6000 S4 PE100). The obtained reads were analyzed following the pipeline outlined in Kolosov et al. (2019).

### RNA-Seq Data Analysis

Paired-end reads were analyzed on the Unix-based server Narval provided by the Digital Research Alliance of Canada. Quality check for raw reads data was performed using FastQC (Andrews 2010). Raw reads were processed through fastp version 0.20.1 which removed adapters, low quality bases (Phred score < 20) and reads shorter than 25 bp from the 3’end (Chen et al. 2018a). Reads were mapped against the *Trichoplusi ni* reference genome GCF_003590095.1 (National Center for Biotechnology Information) using STARv2.7.9a (Dobin et al. 2013; Fu et al. 2018; Liu and Li 2019). FeatureCounts was used to obtain the raw gene counts (Supplemental Table 3). Only genes with expression counts greater than 15 in at least one sample were considered. Differentially expressed genes were identified using DESeq2 assuming a negative binomial (gamma-Poisson) distribution and using a criteria of log_2_-fold change (logFC) (Love et al. 2014). Differentially expressed genes were identified by adjusting for false discovery rate p value (padj) ≤ -0.5 and a fold change that is ≥ 2 or ≤ -2 (Supplemental Table 2). Data visualization was performed using MetaboAnalyst and ExpressAnalyst platforms (Pang et al. 2020; Liu et al. 2023). The raw read data (FASTQ) from this study have been deposited in the NCBI Sequence Read Archive (Bioproject ID #PRJNA1073690).

### Bioinformatic Prediction of Hederagenin Modification by Cytochrome P_450_

Three computational platforms were used to identify the putative site of cytochrome P_450_ oxidation. Way2Drug (http://www.way2drug.com/somp/) uses a machine learning approach incorporating data from known structure–activity relationships to predict the modification of novel substrates (Druzhilovskiy et al. 2017). Xenosite (https://xenosite.org) uses publicly available cytochrome P_450_ metabolism data of over 680 compounds to build neural networks for predictive models (Matlock et al. 2015; Zaretzki et al. 2013; Dang et al. 2020). SMARTCyp (https://smartcyp.sund.ku.dk/mol_to_som) uses the density functional theory determinations to calculate the activation energy of the different residues on the molecule to identify the most reactive atoms (Rydberg et al 2010; Olsen et al. 2019). To identify the putative target site of cytochrome P_450_ enzymes on hederagenin, the simplified molecular input line entry system (SMILES) format for hederagenin was deposited into these different tools.

## Results

### Hederagenin Toxicity and Antifeedant Activity

Fourth instar *T. ni* caterpillars were fed artificial diet containing ethanol or hederagenin (2–80 μg) for 48 h. Mortality was not observed over the hederagenin levels tested.

**Fig. 1:**
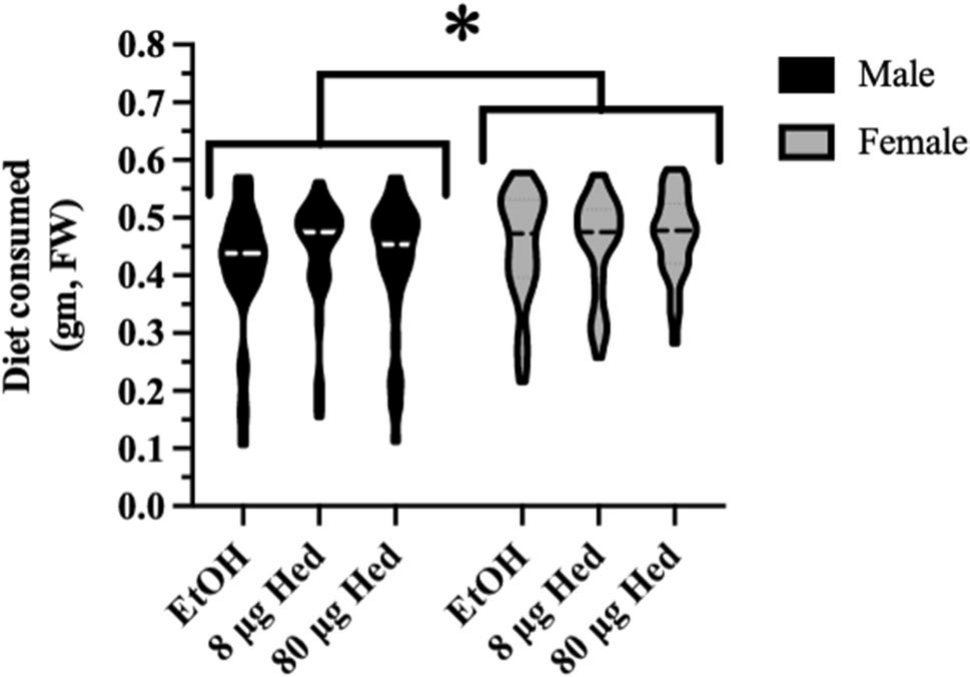
**No choice feeding experiment**. Male and female 4th instar *Trichoplusia ni* caterpillars were fed artificial diet with ethanol (EtOH, control) or the saponin aglycone hederagenin (Hed, 8 or 80 μg). The amount of diet (fresh weight, FW) consumed by caterpillars in 18 h is represented by a violin plot. The dashed line represents the median amount consumed. An asterisk indicates significant differences (*p* ≤ 0.05)

Hederagenin concentration did not affect feeding behaviour of 4^th^ instar caterpillars fed artificial diet containing ethanol or hederagenin (8 μg and 80 μg) for 18 h (Hederagenin: F_(2,218)_ = 0.413; *p* = 0.662) (Fig. 1). However, female caterpillars consumed about 7% more diet than males (Sex: F_(1,218)_ = 5.628; *p* = 0.019).

### Effect of Hederagenin on *Trichoplusia ni* Gene Expression

Caterpillars were allowed to feed on diet for 24 h and then their guts + Malpighian tubules dissected for transcriptomic analysis. For each sample, an average of 35.2 million total 100 bp reads were obtained (Supplemental Table S1). After processing to remove adaptor sequences and poor quality reads, the sample average was 35.3 million reads with a Phred score ≥ 20. The high quality reads were aligned to the *T. ni* genome (NCBI) with a mapping efficiency of 81.9%.

**Fig. 2:**
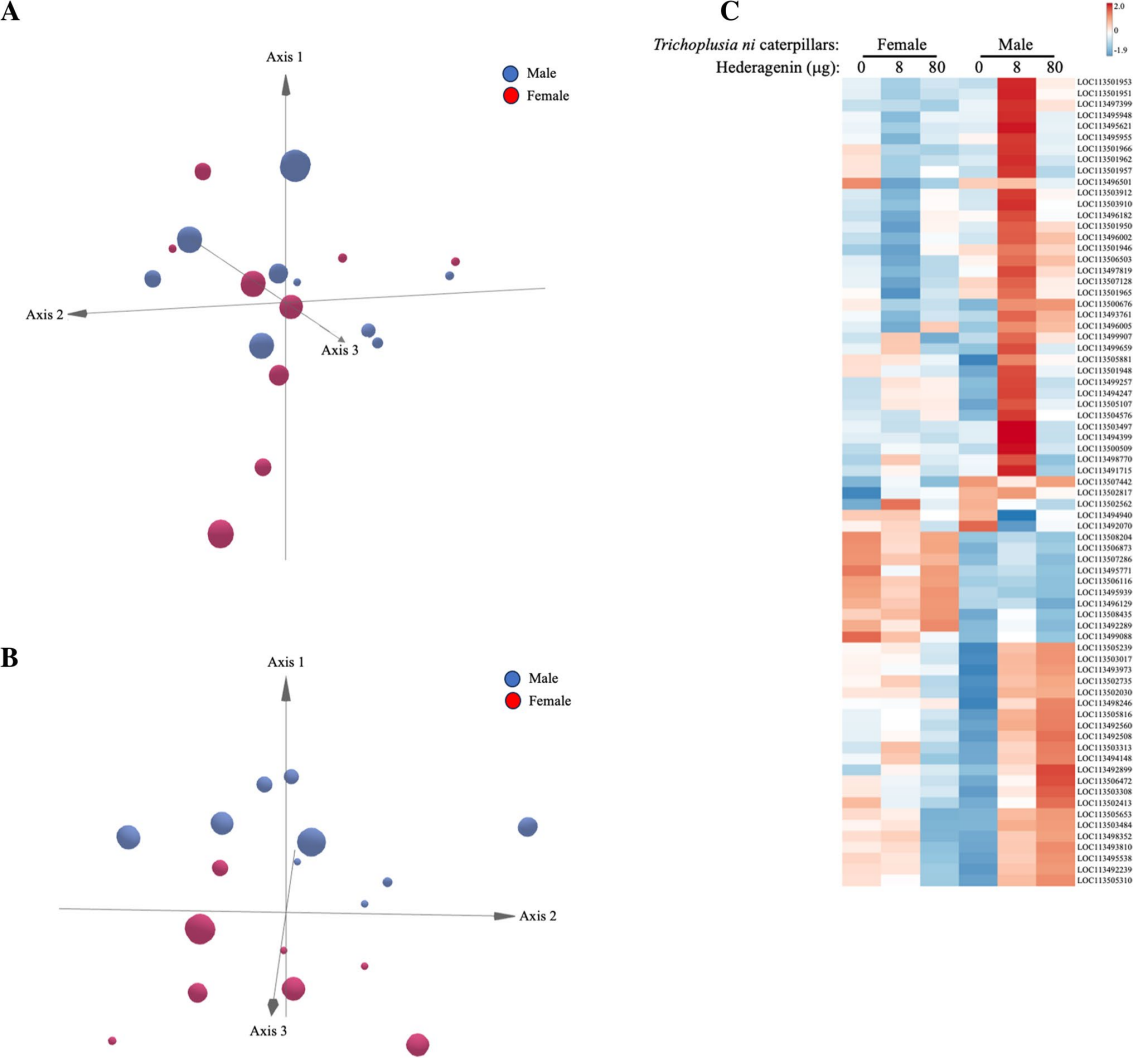
**Gut + Malpighian tubule gene expression of 4**^**th**^ **instar *Trichoplusia ni* caterpillars fed artificial diet with the saponin aglycone hederagenin.** 4^th^ instar caterpillars were fed on diet spiked with ethanol (control) or hederagenin (8 or 80 μg) for 24 h. Total RNA was extracted from dissected gut + Malpighian tubule samples for transcriptomics (RNA-Seq). **A) Principal component analysis (PCA) of gene expression** (Supplemental Table S2). **B) PCA of differentially expressed genes** (Supplemental Table S3). **C) Heat map of differentially expressed genes.** PCA plots depict female (red circles,  ) and male (blue circles,  ) *T. ni* caterpillar gene expression patterns on the EtOH control (small circles) or diet containing 8 μg (medium circles) or 80 μg (large circles) hederagenin. The gene annotations associated with the heat map are found in Supplemental Table S3

**Fig. 3:**
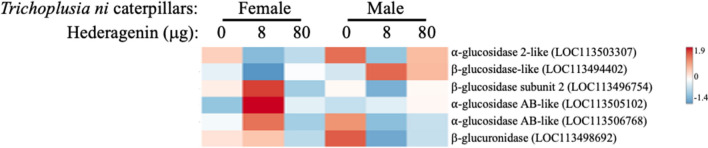
**Expression patterns of genes encoding glycosidase enzymes in *Trichoplusia ni* caterpillars fed artificial diet with the saponin aglycone hederagenin.** Male and female *T. ni* caterpillars were fed diet spiked with ethanol (EtOH; control) or hederagenin (8 or 80 μg) for 24 h. Total RNA was extracted from dissected gut + Malpighian tubule samples for transcriptomics (RNA-Seq). Transcript expression is represented in a heat map. The gene annotations associated with the heat map are found in Supplemental Table S3

In total, 12,109 genes were expressed in *T. ni* gut + Malpighian tubule tissues (Fig. 2A; Supplemental Table 2). As expected, > 99% of the genes were constitutively expressed between treatments and only 73 genes were found to be differentially expressed (Fig. 2B, C; Supplemental Fig. S1, S2, Supplemental Table S3). A number of genes encoding glucosidases (LOC113503307, LOC113494402, LOC113496754, LOC113505102, LOC113506768) and other glycosidases (LOC113504563, LOC113496200, LOC113499077, LOC113495720, LOC113498692, LOC113504733, LOC113506721, LOC113500731, LOC113492303) were expressed (Fig. 3) (Supplemental Table 2).

### Female *Trichoplusia ni* Caterpillars: Effect of Hederagenin on Gene Expression

In female *T. ni* caterpillars fed artificial diet spiked with the hederagenin, there was little difference in gut + Malpighian tubule gene expression compared to the ethanol control. At low hederagenin concentration (8 μg), only two genes were differentially expressed: *uncharacterized LOC113496501* and *lachesin-lik*e (LOC113502562) which are down- and upregulated, respectively, and there was no difference in gene expression in insects fed the higher hederagenin diet (80 μg) (Supplemental Fig. S1A, B, Table S3).

### Male *Trichoplusia ni* Caterpillars: Effect of Hederagenin on Gene Expression

**Fig. 4:**
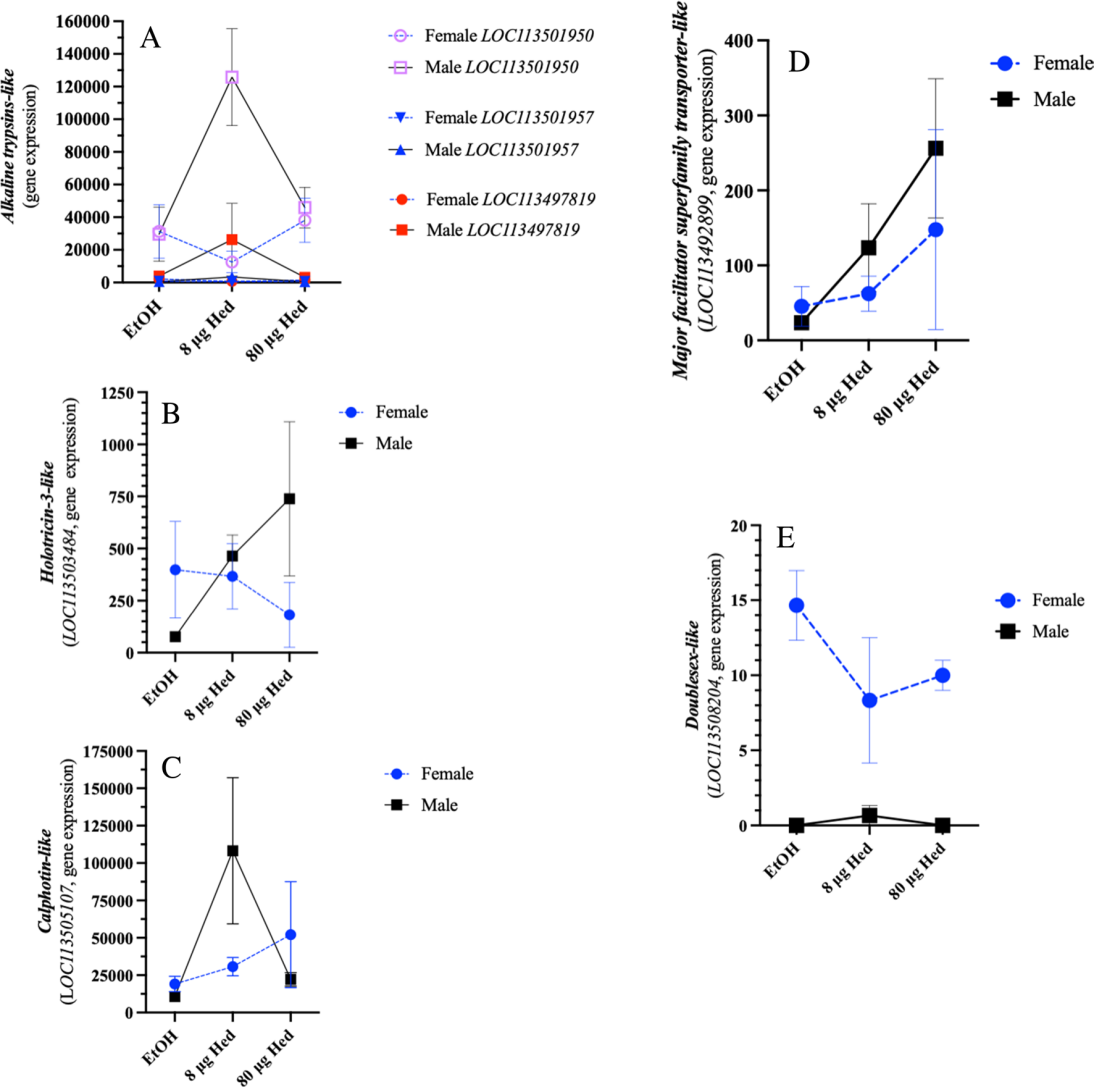
**Sex-specific gene expression of *Trichoplusia ni* caterpillars fed artificial diet with the saponin aglycone hederagenin.** Male (solid line) and female (dashed line) 4^th^ instar *T. ni* caterpillars were fed on diet spiked with ethanol (EtOH; control) or hederagenin (Hed; 8 or 80 μg) for 24 h. Total RNA was extracted from dissected gut + Malpighian tubule samples for transcriptomics (RNA-Seq). Expression of genes higher in male caterpillars **A) Representative *alkaline trypsin-like* genes** (*LOC113501950, LOC501957, LOC113497819*), **B) *Holotricin-3-like*** (likely the homolog **gloverin**-type antimicrobial protein) (*LOC113503384*), **C) *Calphotin-like*** (*LOC113505107*), **D) *Major facilitor superfamily (MFS) transporter-like*** (*LOC113492289*). Expression of genes higher in female caterpillars **E) *Doublesex-like**** (LOC113508204)*

In contrast to female caterpillars, male caterpillars had more differentially expressed genes when fed the hederagenin-spiked diets compared to the ethanol control. At low hederagenin concentration (8 μg); 20 genes were upregulated and 2 genes were downregulated (Supplemental Fig. S1C, Supplemental Table S3). Many of the gut + MT-associated genes upregulated in response to hederagenin were trypsin-like proteins that are involved in protein digestion (differentially expressed trypsin genes: *LOC113500509, LOC113501951, LOC113501953, LOC113501966, LOC113501965, LOC113499659, LOC113501950, LOC113501948, LOC113501957, LOC113501962, LOC113497819, LOC113501946, LOC113503910*) (Fig. 4A).

In males fed on artificial diet spiked with the higher hederagenin concentration (80 μg), 21 genes were upregulated and 1 gene was downregulated (Supplemental Fig. S1D, Supplemental Table S3). In particular, the stress-responsive gene encoding the glycine-rich, antimicrobial peptide putative holotricin-3-like protein (LOC113503484) was upregulated in male caterpillars facing hederagenin challenge (Bulet et al. 1999; Tatić et al. 2023) (Fig. 4B); this is likely a gloverin-type peptide which are found in Lepidoptera and share sequence similarity with coleopteran holotricins. Another stress-responsive gene, *calphotin-like* (*LOC113505107*), was upregulated in male *T. ni* caterpillars fed diet with the lower hederagenin concentration (Fig. 4C). The major facilitator superfamily (MFS) transporter is a single polypeptide transporter involved in the transport of endogenous metabolites or xenobiotics (Reddy et al. 2012). The expression of this gene (*LOC113492899*) increased with hederagenin dosage and in caterpillars fed 8 μg hederagenin, an increase in gene expression is observed in male caterpillars (Fig. 4D).

### Male vs Female *Trichoplusia ni* Caterpillar Gene Expression

Male and female caterpillars showed some distinct gene expression patterns. In particular, genes encoding trypsin (LOC113500509, LOC113501951, LOC113501953, LOC113501966, LOC113501950, LOC113501948, LOC113501957, LOC113501962, LOC113497819, LOC113501946, LOC113503910) were higher in males exposed to hederagenin than females (Fig. 4A, Supplemental Fig. S2. In contrast, a gene encoding doublesex-like (LOC113508204) was more highly expressed in females (Fig. 4E, Supplemental Fig. S2).

**Fig. 5:**
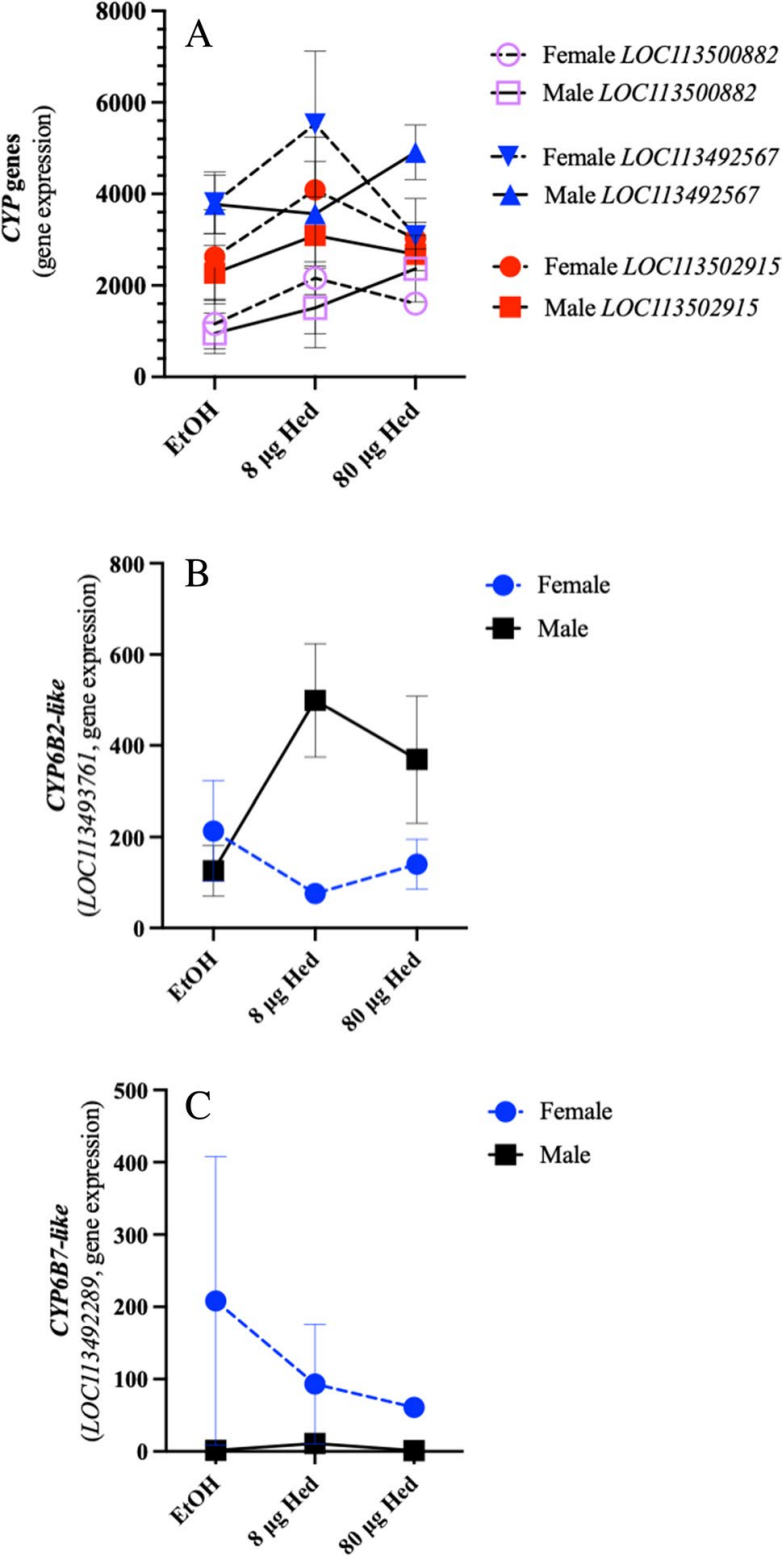
**Cytochrome P**_**450**_** (*CYP450*) gene expression in *Trichoplusia ni* caterpillars fed artificial diet with the saponin aglycone hederagenin.** Male (solid line) and female (dashed line) 4th instar *T. ni* caterpillars were fed diet spiked with ethanol (EtOH; control) or hederagenin (Hed; 8 or 80 μg) for 24 h. Total RNA was extracted from dissected gut + Malpighian tubule samples for transcriptomics (RNA-Seq). **A) Representative *CYP* genes** (*LOC113500882, LOC113492567, LOC113502915*) that do not have a diet- or sex-specific difference, **B) *CYP6B2-like*** (*LOC113493761*), **C) *CYP6B7-like*** (*LOC11349289*)

Regardless of diet or caterpillar sex, most detoxification genes were constitutively expressed (as an example see Fig. 5A); approximately 17 *UDP-glycosyl transferases*, 48 *esterases*, 23 *GST* and 62 *CYP* genes were not affected by hederagenin diet or sex-specific. Many of these genes are likely involved in metabolic processes related to cellular homeostasis and do not necessarily play a detoxification role, but there may be some generic detoxification genes constitutively upregulated when the caterpillar is feeding to enable the insect to cope with the myriad of plant compounds that may be in its diet. However, two genes encoding cytochrome P_450_ 6B7-like (LOC113492289) and cytochrome P_450_ 6B2-like (LOC113493761) showed diet- and/or sex-related expression patterns. Cytochrome P_450_ enzymes are involved in xenobiotic detoxification, particularly those associated with the subfamily 6B (Table 1) (Hlavica 2011). Expression levels of *cytochrome P*_*450*_* 6B7-like* (*CYP6B7-like; LOC113492289*) in female gut + Malpighian tissues were ~ 90 times higher than in male caterpillars (Fig. 5C, Supplemental Fig. S2C). In male caterpillars, transcript expression of *cytochrome P*_*450*_* 6B2-like* (*LOC113493761*) increased in response to hederagenin and was twice as high in males fed artificial diet with 80 μg hederagenin compared to female caterpillars (Fig. 5B, Supplemental Fig. S1A).

**Table 1:** Caterpillar cytochrome P_450_ 6B enzymes potentially involved in the detoxification of plant specialized metabolites (PSMs) or insecticides (I). Differentially expressed genes identified in this study are bolded

Cytochrome P_450_	Caterpillar species	Larval instar and/or tissue expression	Gene expression	Enzyme activity	References
CYP6B1	Black swallowtail, *Papilio polyxenes*	Midgut, fat body	Induced by xanthotoxin, bergapten, angelicin, sphondin, flavone, coumarin, psoralen, xanthotoxol (PSM)	Metabolizes xanthotoxin, psoralen, trioxsalen, isopimpinellin, angelicin, sphenodin, visnagin, khellin, flavone, α-naphthoflavone, coumarin (PSM) and diazinon (I)	Ma et al. 1994; Hung et al. 1995a, b; Petersen et al. 2001; Li et al. 2003; McDonnell et al. 2004; Li et al. 2004; Wen et al. 2005; Wen et al. 2006a, b
**CYP6B2**	**Cabbage looper, *Trichoplusia ni***	**Gut + Malpighian tubule**	**Induced by hederagenin (PSM) in male caterpillars**		**This study**
CYP6B2	Codling moth, *Cydia pomonella*		Higher expression in insecticide (spinosad, deltamethrin, azinphos-methyl)-resistant insects. Knockout lines of this gene are more susceptibile to deltamethrin and azinphos-methyl (I)		Wan et al. 2019; Dai et al. 2022; Idier et al. 2023
CYP6B2	Cotton bollworm, *Helicoverpa armigera*	Midgut	Induced by α-pinene, peppermint oil, 2-heptanone, *cis*-3-hexenyl acetate, limonene, nerolidol, flavone, coumarin, 2,4-dihydroxy-7-methoxy-1,4-benzoxazin-3-one, visnagan (PSM), deltamethrin, phenobarbital (I)		Wu et al. 1995; Zhou et al. 2010; Chen et al. 2019; Wu et al. 2021
CYP6B3	Black swallowtail, *Papilio polyxenes*	Midgut	Induced by xanthotoxin, bergapten, angelicin, sphondin (PSM)	Metabolizes xanthotoxin, psoralen, trioxsalen, isopimpinellin, angelicin, spenodin, visnagin, khellin, α-naphthoflavone (PSM)	Ma et al. 1994; Hung et al. 1995a, b; Petersen et al. 2001; Wen et al. 2005; Wen et al. 2006a, b
CYP6B4	Eastern tiger swallowtail, *Papilio glaucus*			Metabolizes angelicin, trioxsalen, psoralen, xanthotoxin, bergapten (PSM)	Li et al. 2003
CYP6B5	Codling moth, *Cydia pomonella*		Higher expression in insecticide (spinosad)-resistant insects		Idier et al. 2023
CYP6B6	Cotton bollworm, *Helicoverpa armigera*	Constitutive in caterpillars	Quercetin (PSM)-induced expression results in increased resistance to λ-cyhalothrin (pyrethroid) (I). Induced by 2-heptanone, *cis*-3-hexenyl acetate, limonene, nerolidol, flavone, coumarin, 2,4-dihydroxy-7-methoxy-1,4-benzoxazin-3-one, visnagan (PSMs), deltamethrin (I)	Metabolizes esfenvalerate (pyrethroid) (I)	Zhou et al. 2010; Zhang et al. 2013; Tian et al. 2017; Chen et al. 2018b; Chen et al. 2019; Shi et al. 2021; Wu et al. 2021
**CYP6B7**	**Cabbage looper, *Trichoplusia ni***	**Gut + Malpighian tubule; higher expression in female caterpillars**			**This study**
CYP6B7	Cotton bollworm, *Helicoverpa armigera*	4th instar caterpillars, midgut	Higher expression in pesticide (deltamethrin, α-cypermethrin, β-cyfluthrin, fenvalerate (pyrethroids))-resistant insects Induced by α-pinene, xanthotoxin, 2-heptanone, *cis*-3-hexenyl acetate, limonene, nerolidol, flavone, coumarin, 2,4-dihydroxy-7-methoxy-1,4-benzoxazin-3-one, visnagan (PSM), deltamethrin, phenobarbital, fenvelerate, phoxim, indoxycarb (I)	Metabolizes esfenvalerate, bifenthrin, fenvalerate, chlorpyrifos (pyrethroids) (I)	Ranasinghe and Hobbs 1998; Ranasinghe et al. 1998; Ranasinghe and Hobbs 1999; Zhang et al. 2010; Zhou et al. 2010; Abd El-Latif, et al. 2014; Zhao et al. 2017; Chen et al. 2019; Huang et al. 2021; Shi et al. 2021; Wu et al. 2021
CYP6B8	Cotton bollworm, *Helicoverpa armigera*	Midgut	Quercetin (PSM)-induced expression results in increased resistance to λ-cyhalothrin (pyrethroid) (I). Induced by xanthotoxin, flavone, chlorogenic acid (PSM), α-cypermethrin, aldrin, diazinon (I)	Metabolizes xanthotoxin, quercetin, flavone, chlorogenic acid, indole-3-carbinol, rutin (PSM) and diazinon, α-cypermethrin and aldrin (I)	Li et al. 2002; Li et al. 2004; Chen et al. 2018b
CYP6B8	Cotton bollworm, *Helicoverpa zea*		Induced by xanthotoxin,		Li et al. 2000
CYP6B17	Eastern tiger swallowtail, *Papilio glaucus* Canadian tiger swallowtail, *Papilio canadensis*			Metabolizes angelicin, trioxsalen, psoralen, xanthotoxin, bergapten (PSM)	Li et al. 2003
CYP6B21	Eastern tiger swallowtail, *Papilio glaucus*			Metabolizes angelicin, trioxsalen, psoralen, xanthotoxin, bergapten (PSM)	Li et al. 2003
CYP6B33	Two-tailed swallowtail, *Papilio multicaudata*		Xanthotoxin (PSM)-induced	Metabolizes angelicin, trioxsalen, psoralen, xanthotoxin, bergapten (PSM)	Mao et al. 2007
CYP6B37	Two-tailed swallowtail, *Papilio multicaudata*		Xanthotoxin (PSM)-induced		Mao et al. 2007
CYP6B38	Fall armyworm, *Spodoptera frugiperda*	Midgut, Malpighian tubules, fat body			Giraudo et al. 2015
CYP6B39	Fall armyworm, *Spodoptera frugiperda*	Midgut, Malpighian tubules	Induced by indole, indole-3-carbinol, xanthotoxin (PSM)		Giraudo et al. 2015
CYP6B40	Fall armyworm, *Spodoptera frugiperda*	Midgut, Malpighian tubules, fat body	Induced by indole, indole-3-carbinol, xanthotoxin (PSM)		Giraudo et al. 2015
CYP6B42	Fall armyworm, *Spodoptera frugiperda*	Midgut, Malpighian tubules, fat body			Giraudo et al. 2015
CYP6B47	Tobacco cutworm, *Spodoptera litura*		Lead-induced expression of *CYP6B47* results in increased resistance to α-cypermethrin and fenvalerate (I)		Zhou et al. 2012a, b
CYP6B50	Fall armyworm, *Spodoptera frugiperda*	Midgut			Giraudo et al. 2015

### Computational Identification of the Potential Site of Cytochrome P_450_-Mediated Hederagenin Modification

**Fig. 6:**
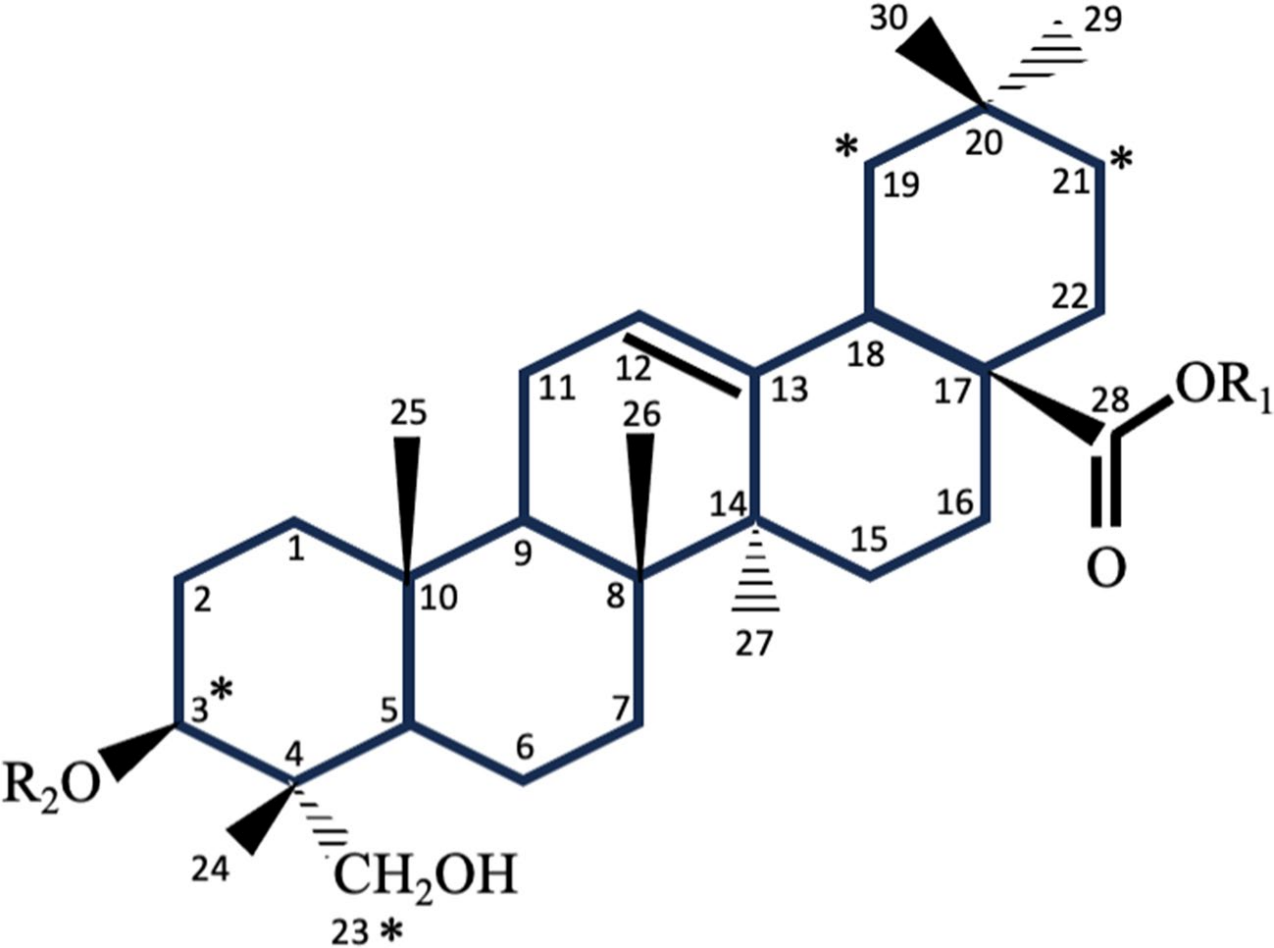
**Structure of the saponin aglycone hedragenin**. The triterpenoid aglycone hederagenin (R1 = H, R2 = H) is typically glycosylated at position C3 or C28. Asterisks indicate putative sites of saponin modification by cytochrome P_450_s identified using bioinformatic tools (Xenosite, Way2Drug and SMARTCyp) (Supplemental Table S4)

Computational platforms designed to identify drug target sites by human cytochrome P_450_ enzymes were used to identify putative site(s) of modification on hederagenin. The sites most consistently identified for oxygenation of hederagenin by cytochrome P_450_ were C23, C3, C19 and C21 (Fig. 6, Supplemental Table S4). However, it must be considered that these platforms were developed to identify the targets of human enzymes rather than insect cytochrome P_450_s. However, the consistent ranking does suggest that these carbons may be promising sites of modification.

## Discussion

At the concentrations tested, hederagenin was not toxic to *T. ni* caterpillars. As well, the aglycone hederagenin did not have an antifeedant role contrary to other studies that identified the saponin 3-*O*-[*O*-β-D-glucopyranosyl-(1➙4-β-D-glucopyranosyl]-hederagenin as a feeding deterrent for 3rd instar *P. xylostella* caterpillars (Shinoda et al. 2002), which was confirmed by Liu et al (2019) who showed that hederagenin monoglucosides reduced *P. xylostella* and *M. sexta* feeding by 90%. This may reflect the difference between the aglycone and the saponin or that *M. sexta* and *P. xylostella* are specialists on Solanaceous and Brassicaceous plants, respectively, while in our study, we used *T. ni* caterpillars, considered an adaptive specialist of Brassicaceous plants. As some plants in the Brassicacea, for example in the genus *Barbarea*, contain saponins as defensive compounds (Shinoda et al. 2002; Hussain et al. 2019), *T. ni* caterpillars likely have strategies, such as detoxification enzymes, that allow them to feed on a plant diet containing hederagenin-type saponins.

Once the saponin is ingested, plant- or insect gut-derived glycosidases likely hydrolyze the sugar ester bond to release the sapogenin (Terra et al. 2019; Lacchini et al. 2023). We identified 14 genes encoding glucosidases and other glycosidases expressed in *T. ni* midgut-Malpighian tubule tissue (Fig. 3, Supplemental Table S2). However, it is unclear which of these enzymes are able to hydrolyze the sugar ester bond of saponins (Liu et al. 2017; Terra et al. 2019). Though in the extremely alkaline midgut of caterpillars (Dow, 1992), some saponins may be labile which may spontaneously lead to their hydrolysis to the sapogenin and sugar.

In response to hederagenin-spiked diet, extremely few genes were differentially regulated in female caterpillars (Supplemental Fig. S1A, B, Supplemental Table S3). In comparison, > 40 genes were upregulated in response to hederagenin in male caterpillars (Fig. 2C, Supplemental Fig. S1C, D, Supplemental Table S3). Both male and female caterpillars had high expression levels of transcripts that encode proteases (i.e. trypsin, chemotrypsin etc.). In addition, a broader expression of genes encoding the serine protease trypsin was observed in male caterpillars (Fig. 4A). The reason for this is unclear. However, in the aquatic microcrustacean *Daphnia sinensis*, genes that encode a number of serine proteases, including 5 *trypsin* genes, are expressed more highly in males (Wang et al. 2022). Saponins can nonspecifically interact and inhibit digestive enzymes (Ishaay, 1986); this increase in *trypsin* expression may be a mechanism to counteract this. However, it is unknown if sapogenins, such as hederagenin, have this property.

Moreover, in male caterpillars, the expression of genes encoding stress-related proteins were observed, for example calphotin-like (LOC113505107) (Fig. 4C) (Wang et al. 2021; Brumin et al. 2020; Pingault et al. 2022; Mahanta et al. 2022; Tatić et al. 2023; Mulla and Tamhane 2023). Even though in *Drosophila melanogaster*, this Ca^2+^-binding protein plays a role in Ca^2+^ buffering in photoreceptors (Weiss et al. 2012), calphotin in other insect Orders appears to be regulated by stress. This gene was downregulated in *H. armigera* caterpillars fed the plant protein defensin and also in *P. xylostella* caterpillars infected with the entomopathogenic fungi *Beauveria bassiana* (Wang et al. 2021; Mulla and Tamhane 2023). In our study, *calphotin-like* (*LOC113505107*) was upregulated in male *T. ni* caterpillars exposed to the lower hederagenin dose (Fig. 4C), which supports other studies which found that this gene was upregulated in Western corn root, *Diabrotica virgifera virgifera*, larvae exposed to Bt-corn, thrips *Thrips palmi* infected by groundnut bud necrosis virus, rickettsia-infected whitefly *Bemisia tabaci*, (Brumin et al. 2020; Mahanta et al. 2022; Pingault et al. 2022), which suggests that these homologues have broader functions in stress responses likely related to their Ca^2+^ binding activity.

Female caterpillars had higher expression of *doublesex-like* (Fig. 4E). Doublesex is involved in sex-differentiation and different alternative splice variants are seen in males *vs* females (Suzuki et al. 2003).

Female caterpillars ate more diet, regardless of hederagenin levels, than males (Fig. 1). This may be because female caterpillars, compared to males, must accumulate sufficient resources at the larval stage for egg production as adults (Wheeler 1996; Wheeler et al. 2000). It also means that females are exposed to higher levels of plant chemical defenses, in the case of our study hederagenin.

Female caterpillars had high expression levels of the gene encoding cytochrome P_450_ 6B7-like protein (LOC113492289) (Fig. 5C). Cytochrome P_450_ enzymes are involved in phase I detoxification, catalyzing the oxygenation of xenobiotic to facilitate their excretion or provide sites for phase II reactions, such as the addition of glutathione by glutathione *S*-transferases (GSTs) (Feyereisen 2012; Esteves et al. 2021; Amezian et al. 2021). Lepidopterans have superfamilies of detoxification genes (i.e. cytochrome P_450_s, GSTs, carboxylesterases) that may reflect their herbivorous lifestyle exposing them to a diversity of plant specialized metabolites, but this also makes them adept at insecticide detoxification leading to pesticide resistance. The *T. ni* genome contains 108 genes that encode cytochrome P_450_ enzymes compared to 90 in the Brassicacea specialist *P. xylostella* and 84 in the mulberry specialist *Bombyx mori* (You et al. 2013; Fu et al. 2018). Most of detoxification-related transcripts were constitutively expressed (for example Fig. 5A); a sex-specific difference in the expression of two *CYP6B* genes (LOC113493761 and LOC113492289) was observed (Fig. 5B, C), highlighting the importance of members of the cytochrome P_450_ 6B family in plant specialized metabolite and insecticide detoxification (Table 1) (Hlavica 2011; Lu et al. 2021; Nauen et al. 2022). Expression of *CYP6B7-like* (*LOC113492289*) was ~ 90% higher in female insects than males (Fig. 5C). In comparison, *CYP6B2-like* (*LOC113493761*) was induced in males feeding on hederagenin-containing diet (Fig. 5B). Though not definitive, computational tools identified a few select carbons as possible sites for cytochrome P_450_ modification of hederagenin (Fig. 6, Supplemental Table S4).

Another interesting gene expressed in gut + Malpighian tubules encodes a major facilitator superfamily-like transporter (LOC113492899). The major facilitator superfamily (MFS) transporter is a single polypeptide transporter involved in the transport of endogenous metabolites or xenobiotics (Reddy et al. 2012). In the generalist two-spotted spider mite, *Tetranychus urticae*, strong upregulation of genes in this family was associated with pesticide resistance or exposure to plant specialized metabolites (Dermauw and Leeuwen 2014). Even though this gene was differentially expressed in male caterpillars exposed to low hederagenin, in both males and females, gene expression increased with dosage suggesting that this transporter may be involved in hederagenin and/or hederagenin-derivative transport (Fig. 4D).

The host plants that herbivorous caterpillars feed upon reflect their ability to detoxify the defensive specialized metabolites found in those plants (Calla et al. 2017). Caterpillars of *T. ni* that feed on plants of the Brassicacea genus *Barbarea* are exposed to hederagenin-type saponins. This may explain why the sapogenin hederagenin is not toxic or an antifeedant to *T. ni* caterpillars. What is striking is that female caterpillars ate 7% more diet than males, implying that, in nature, they may be exposed to more noxious plant compounds than males (Fig. 1). This was reflected in the detoxification-related gene expression where *CYP6B7-like* (*LOC113492289*) expression was higher in female caterpillars or *CYP6B2-like* (*LOC113493761*) which was induced in response to hederagenin in males (Fig. 5B, C). However, detoxification enzymes not only metabolize plant specialized metabolites but also other xenobiotics such as insecticides. Indeed, sex-linked resistance has been identified in a number of insect species (examples: Heckel et al 1998; Kanga et al. 2001; Reyes and Sauphanor 2008; Banazeer et al. 2022). Thus, identifying sex-specific detoxification genes and understanding their links to resistance strategies has important implications for pesticide management and sustainable agricultural practices.

### Supplementary Information

Below is the link to the electronic supplementary material.

Supplementary file1 (PNG 112 KB)

Supplementary file2 (PNG 438 KB)

Supplementary file3 (PNG 233 KB)

Supplementary file4 (PNG 109 KB)

Supplementary file5 (XLSX 1541 KB)

## Data Availability

Raw read data (FASTQ) has been deposited to the NCBI Sequence Read Archive (Bioproject ID#PRJNA1073690).

## Abbreviation

DEGs: Differentially expressed genes
