# Supplementary figures and images for: Trichoplusia ni Transcriptomic Responses to the Phytosaponin Aglycone Hederagenin: Sex-Related Differences
Source: J Chem Ecol. 2024 Mar 5;50(3-4):168–84. doi: 10.1007/s10886-024-01482-1 (PMC11041752; doi:10.1007/s10886-024-01482-1)

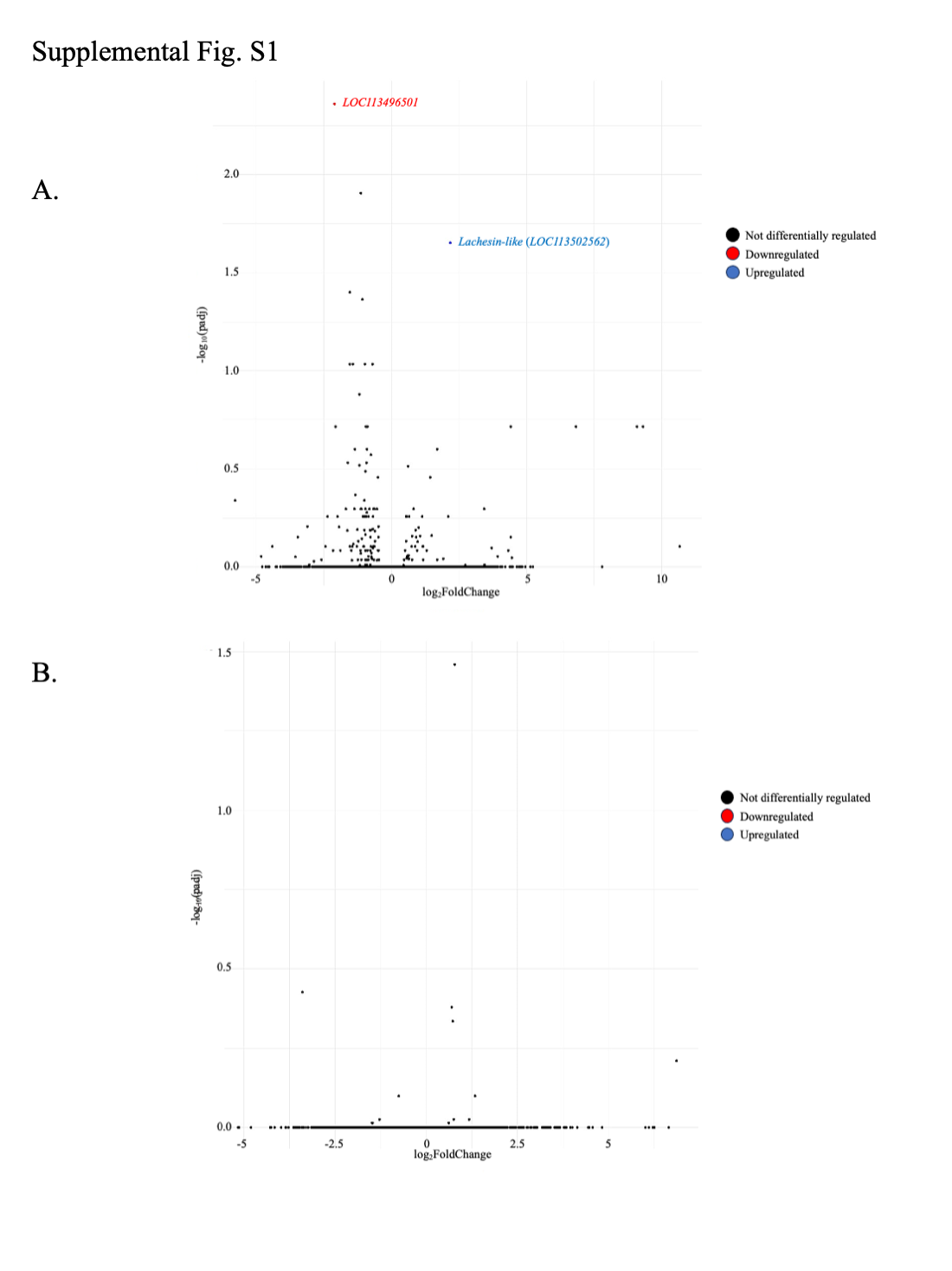

Supplement: Supplementary file 1 — Supplementary file1 (PNG 112 KB) [file 10886_2024_1482_MOESM1_ESM.png]

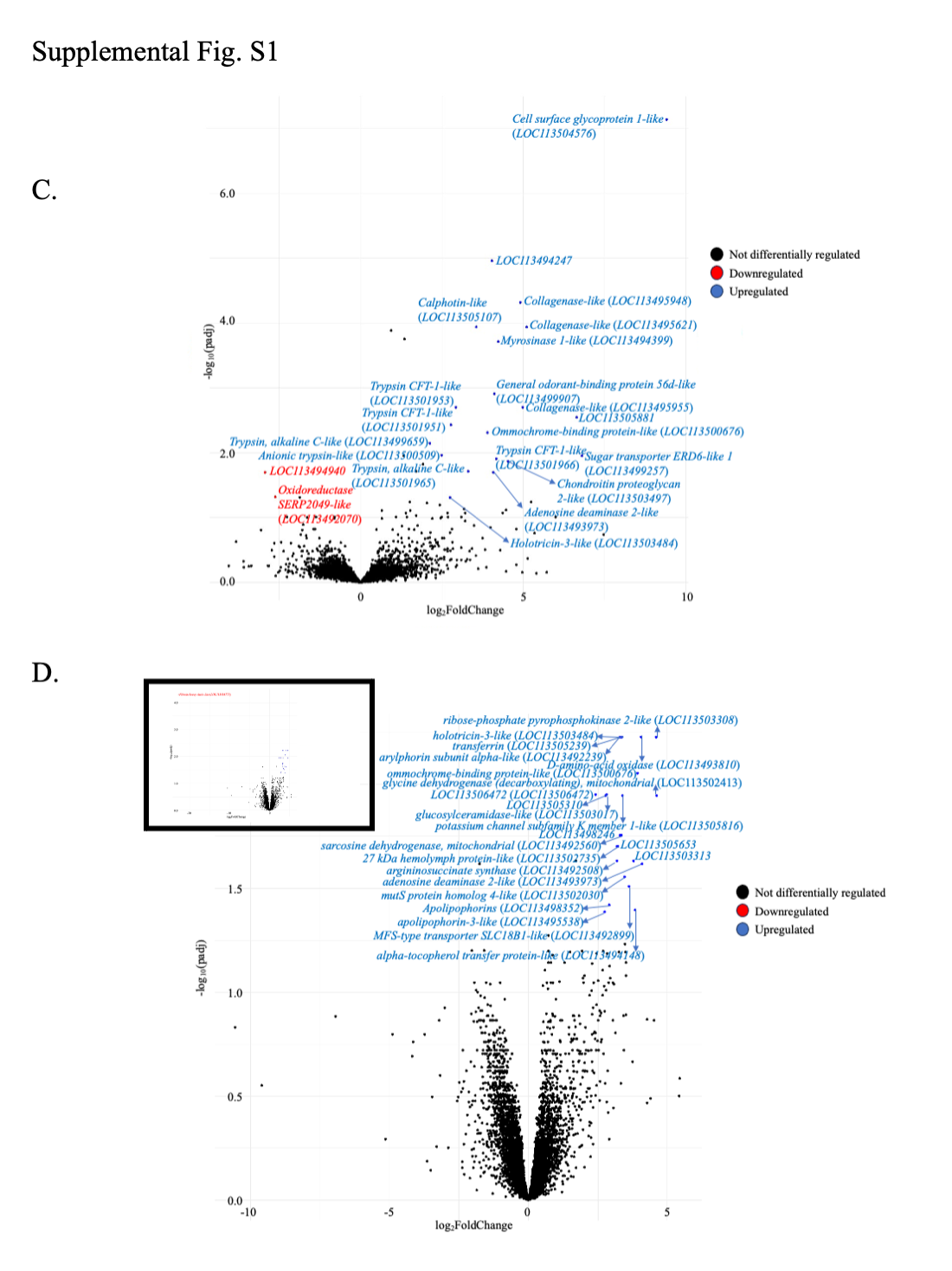

Supplement: Supplementary file 2 — Supplementary file2 (PNG 438 KB) [file 10886_2024_1482_MOESM2_ESM.png]

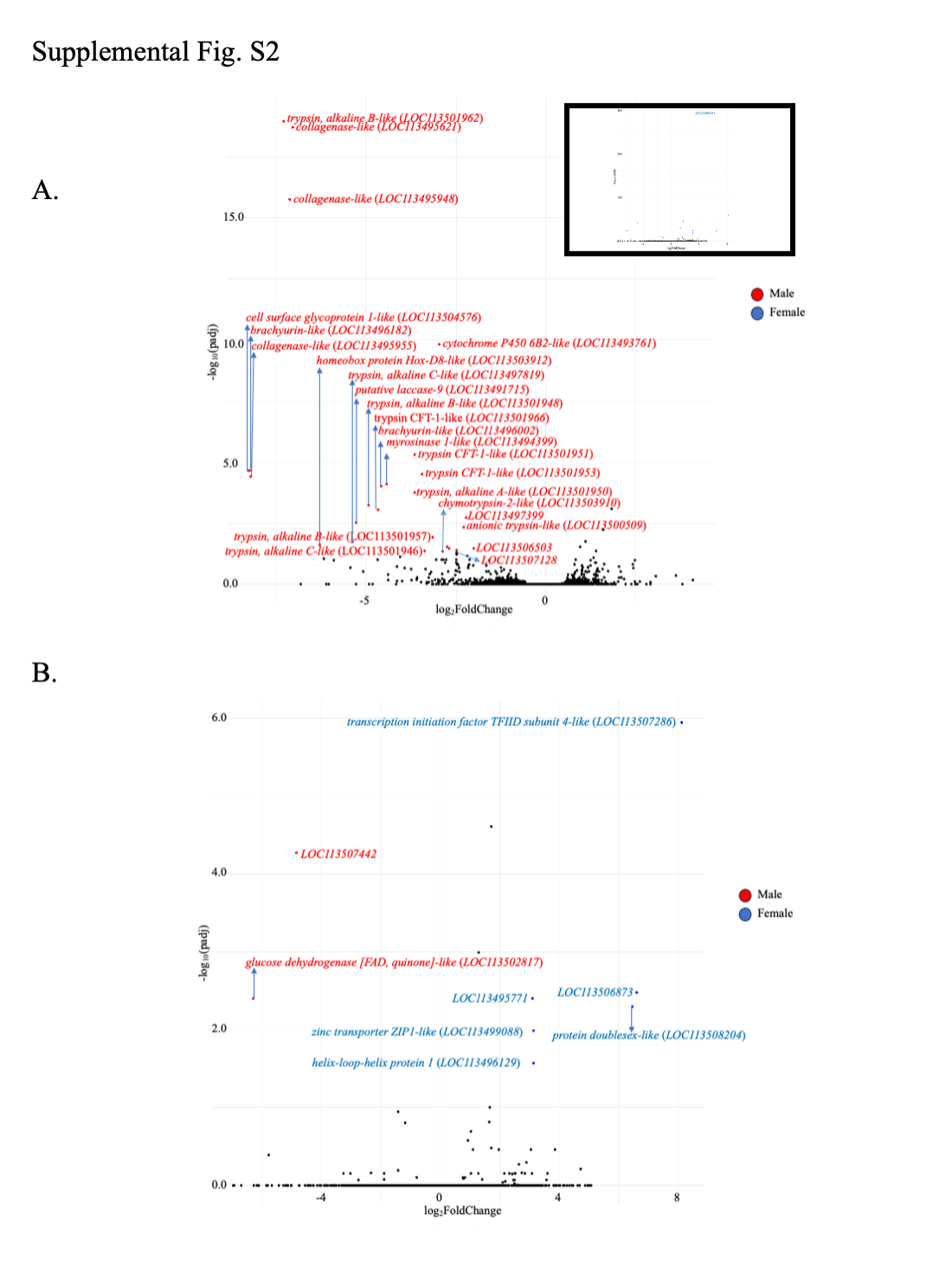

Supplement: Supplementary file 3 — Supplementary file3 (PNG 233 KB) [file 10886_2024_1482_MOESM3_ESM.png]

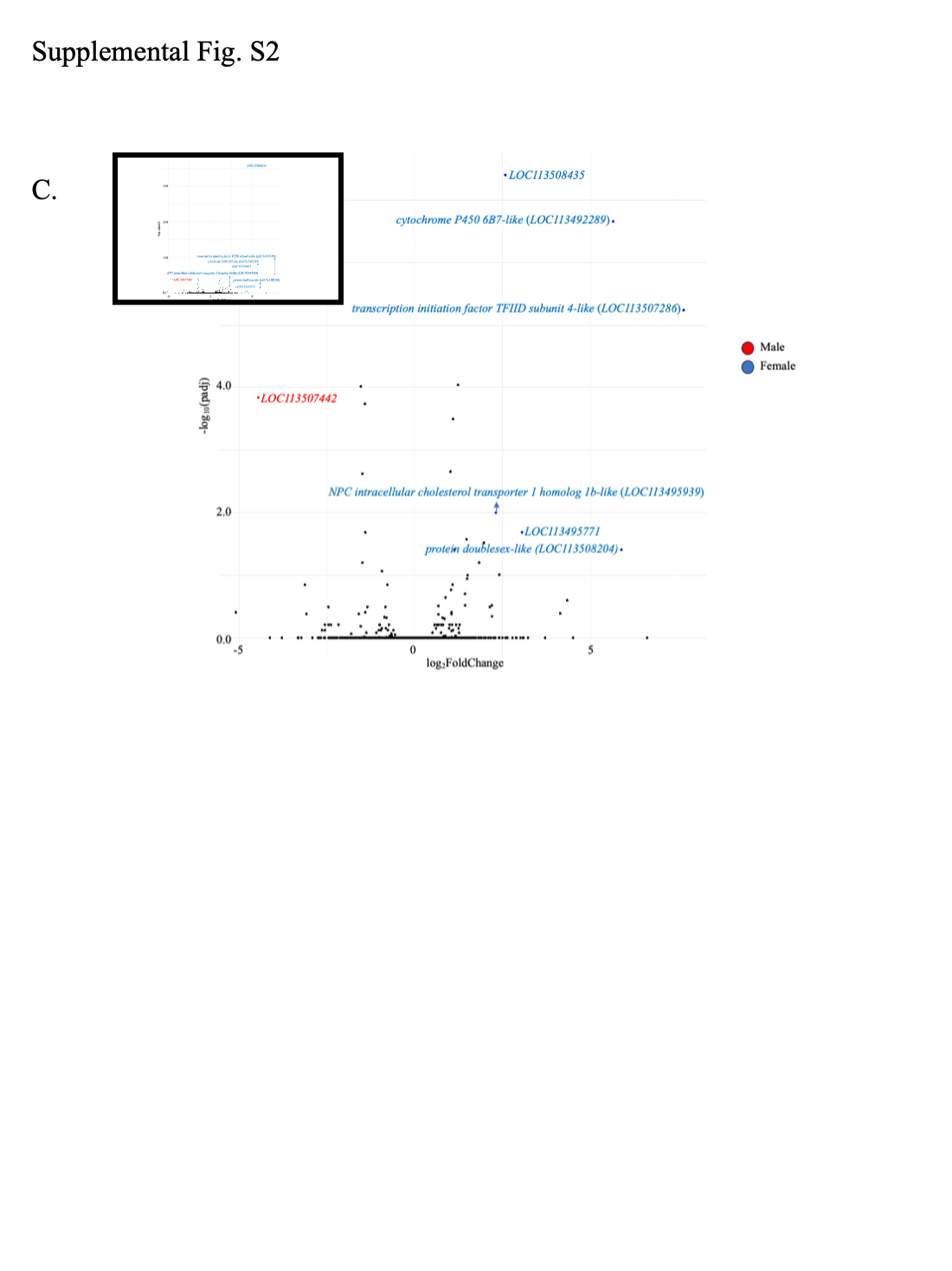

Supplement: Supplementary file 4 — Supplementary file4 (PNG 109 KB) [file 10886_2024_1482_MOESM4_ESM.png]
